# Comparative Studies on Thermal Decompositions of Dinitropyrazole-Based Energetic Materials

**DOI:** 10.3390/molecules26227004

**Published:** 2021-11-19

**Authors:** Jing Zhou, Chongmin Zhang, Huan Huo, Junlin Zhang, Zihui Meng, Tao Yu, Yingzhe Liu, Xiaolong Fu, Lili Qiu, Bozhou Wang

**Affiliations:** 1 School of Chemistry and Chemical Engineering, Beijing Institute of Technology, Beijing 102488, China; zhoujing19872006@163.com (J.Z.); mengzh@bit.edu.cn (Z.M.); 2 Xi’an Modern Chemistry Research Institute, Xi’an 710065, China; iceand010@163.com (C.Z.); huohuan-234@163.com (H.H.); junlin-111@163.com (J.Z.); fischer@wo.cn (T.Y.); liuyz_204@163.com (Y.L.)

**Keywords:** dinitropyrazole, mechanism, ReaxFF force field, thermal decomposition, trimerization

## Abstract

Dinitropyrazole is an important structure for the design and synthesis of energetic materials. In this work, we reported the first comparative thermal studies of two representative dinitropyrazole-based energetic materials, 4-amino-3,5-dinitropyrazole (LLM-116) and its novel trimer derivative (LLM-226). Both the experimental and theoretical results proved the active aromatic N-H moiety would cause incredible variations in the physicochemical characteristics of the obtained energetic materials. Thermal behaviors and kinetic studies of the two related dinitropyrazole-based energetic structures showed that impressive thermal stabilization could be achieved after the trimerization, but also would result in a less concentrated heat-release process. Detailed analysis of condensed-phase systems and the gaseous products during the thermal decomposition processes, and simulation studies based on ReaxFF force field, indicated that the ring opening of LLM-116 was triggered by hydrogen transfer of the active aromatic N-H moiety. In contrast, the initial decomposition of LLM-226 was caused by the rupture of carbon-nitrogen bonds at the diazo moiety.

## 1. Introduction

Thermal property is a key factor for the application of energetic materials [1,2,3,4], which is related to safety production, transportation and storage. Unlike traditional energetic structures with carbonaceous backbones, a large part of modern energetic materials are designed based on poly nitrogen-rich heterocycles constructed through the coupling of various heterocyclic units [5,6,7,8]. From structural standpoint, both the heterocyclic units and their coupling manner can significantly influence the thermal properties of the obtained coupling products [9]; however, the detailed effect and mechanism of the influence still remains unclear since little comparative thermal research was reported.

**Figure 1:**
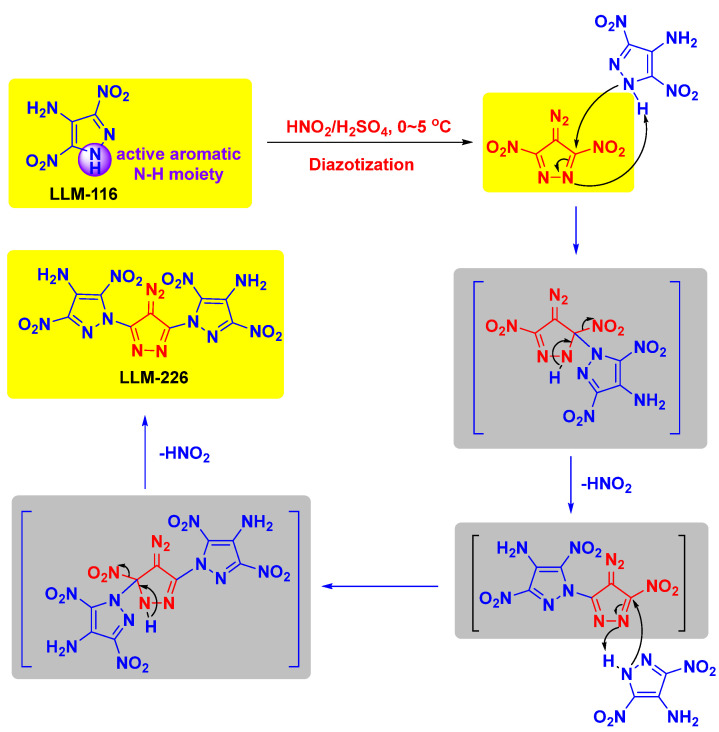
The synthesis of LLM-226 based on self-coupling of LLM-116.

Active aromatic N-H moiety widely exists in energetic structures and causes incredible variations in the physicochemical characteristics and detonation performances of corresponding energetic materials [10,11,12]. With a highly acidic aromatic N-H bond, 4-amino-3,5-dinitropyrazole [13,14] (LLM-116) has been successfully applied as an active nucleophile in the design and synthesis of other heterocycle-based energetic materials [15,16]. 4-Diazo-3,5-bis(4-amino-3,5-dinitropyrazol-1-yl)pyrazole (LLM-226) is a novel trimer structure of LLM-116 which was recently beautifully prepared through a self-coupling reaction [17]. (Figure 1) Compared with LLM-116, the trimerization retains the pyrazole backbones but removes the active H from their aromatic N-H moieties. Due to the abundant nitro and amino groups, both LLM-116 and LLM-226 are promising candidates for insensitive high explosives, with their energetic properties as follows [17]: density of 1.90 and 1.83 g·cm^−^^3^; Dh_50_ (2.5 kg weight) of 177 and 31cm; friction sensitivity (BAM) of 0/10 @ 36kg and 1/10 @ 32.4kg; Spark Sensitivity (J @ 0 Ohms) of 0.038 and 0.014; detonation velocity (calculated) of 8497 and 8220 m/s; detonation pressure (calculated) of 31.89 and 28.0 Gpa; enthalpy of formation (calculated) of 221.1 and 686.63 kJ/mol. Meanwhile, the novel structures and self-coupling manner make these dinitropyrazole derivatives ideal for comparative thermal studies to clarify how the heterocyclic units and coupling manners will affect corresponding thermal properties. Herein, a systematic comparative thermal research on LLM-116 and LLM-226 were carried out through both experimental and theoretical approaches. Their thermal behaviors, thermolysis kinetics, as well as thermal decomposition mechanism were all investigated and compared to get a deep understanding on the pyrolysis processes of the two related dinitropyrazole-based energetic materials.

## 2. Results and Discussion

The strong intramolecular and intermolecular hydrogen bonding interactions between the amino and nitro groups [18,19] give LLM-116 high crystal density and superior insensitivities to mechanical stimulations. Compared with LLM-116, LLM-226 possesses similar hydrogen bonding interactions and a larger conjugate system. Although the expansion of the aromatic conjugate structure can improve the thermal stability of LLM-226, the newly formed diazo structure may bring complex influence on its thermal behaviors. On the one hand, most diazo structures suffer poor stability [20,21] and will lose dinitrogen to afford more stable structures under heating conditions. On the other hand, the introduction of salt structures, especially inner salt structures, has been proved as an efficient approach to improve the thermal stabilities of energetic materials [22,23].

**Figure 2:**
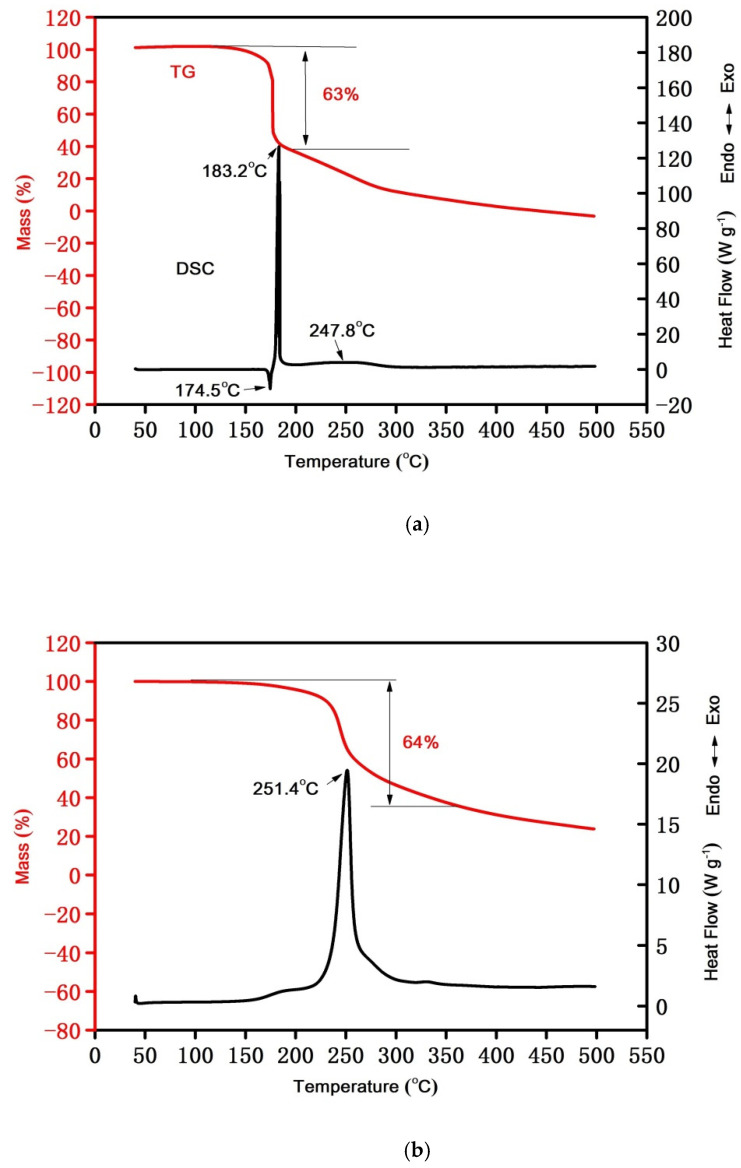
DSC-TG curves of LLM-116 (**a**) and LLM-226 (**b**).

The detailed thermal decomposition behaviors of the two energetic structures were first investigated and compared through the DSC-TG method. (Figure 2) As shown in the DSC curve, the major decomposition process of LLM-116 started shortly after its melting. The major exothermic peak at the heating rate of 10 °C·min^−1^ was observed at 183 ^o^C in a sharp peak shape. A small and broad exothermic peak was found with the peak temperature at around 247 °C, which may be caused by the interactions between the decomposition products of LLM-116. Its major weight loss was an intensive peak that corresponded with the major exothermic peak in DSC. Compared with LLM-116, the major decomposition peak temperature of LLM-226 was much higher and the major exothermic peak shape was much broader. No melting process was observed before the thermal decomposition process of LLM-226, and its weight loss process lasted in a wide temperature range. Apparently, the active aromatic N-H moiety significantly influenced the thermal decomposition of LLM-116; in contrast, the larger aromatic conjugate structure of LLM-226 provided an additional stabilizing effect but also led to less concentrated exothermic behaviors.

**Figure 3:**
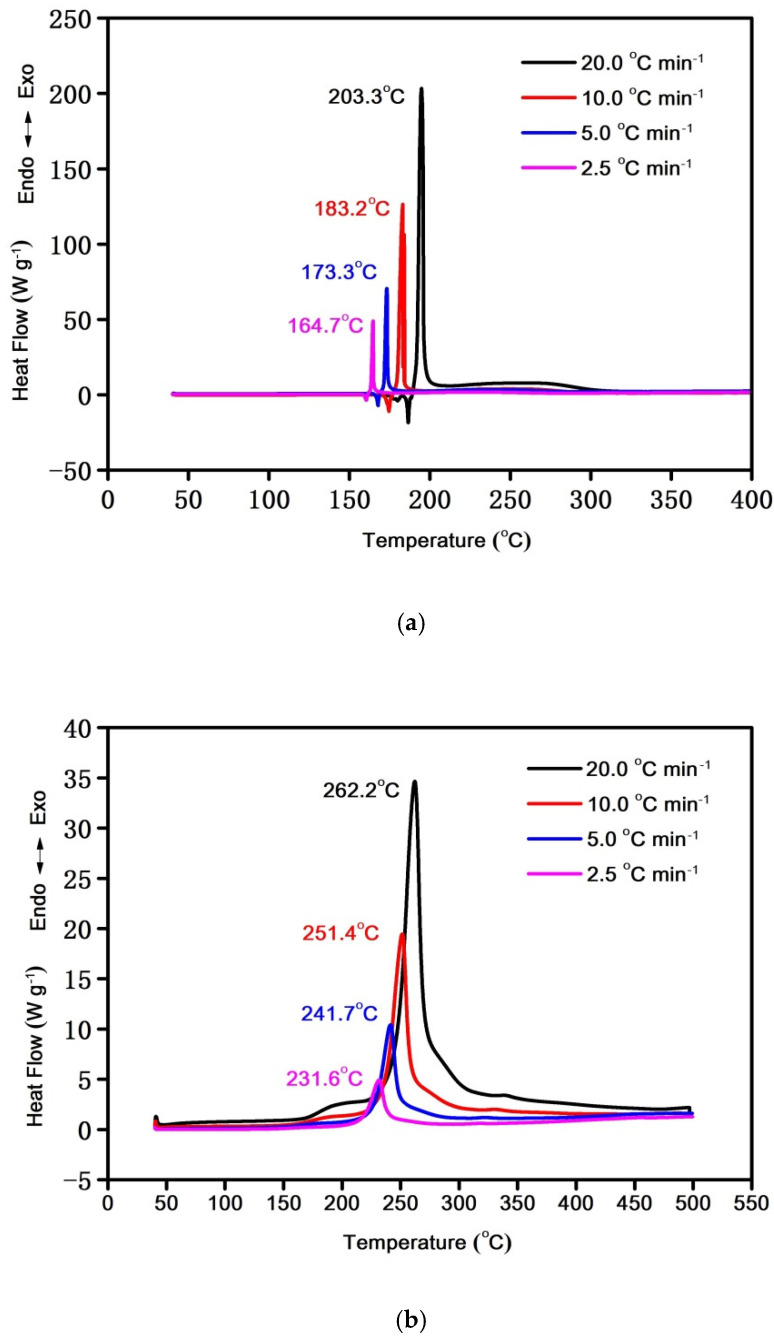

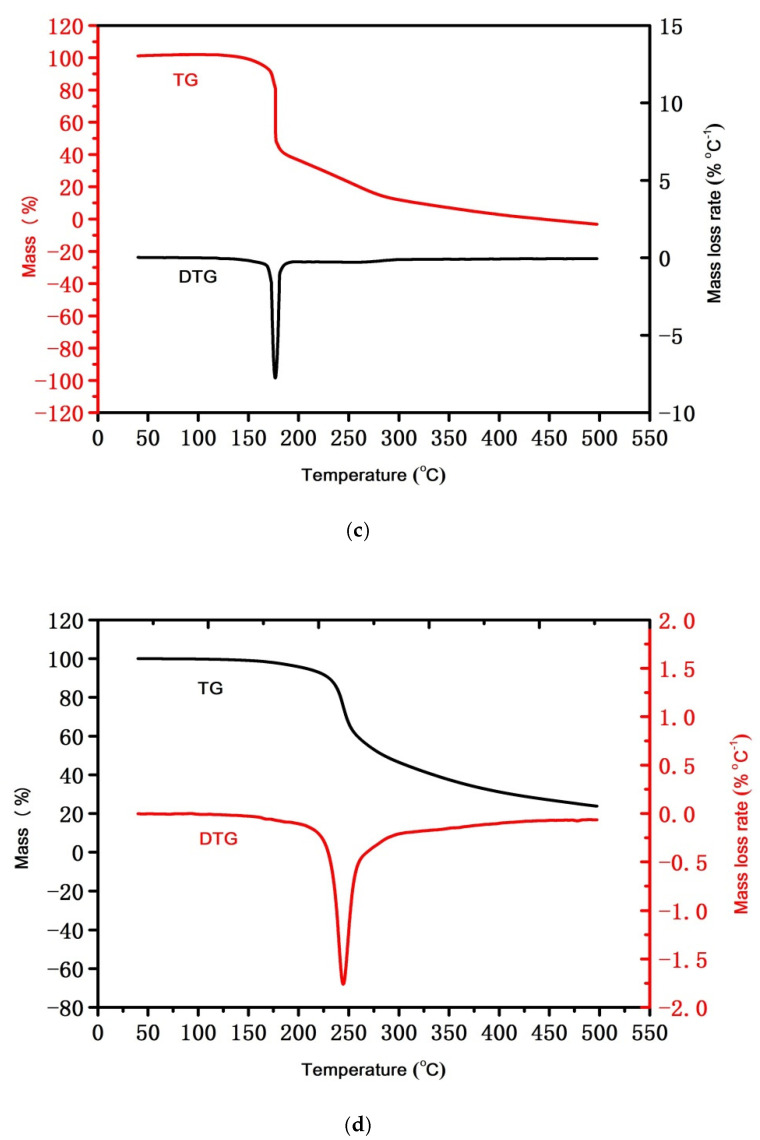

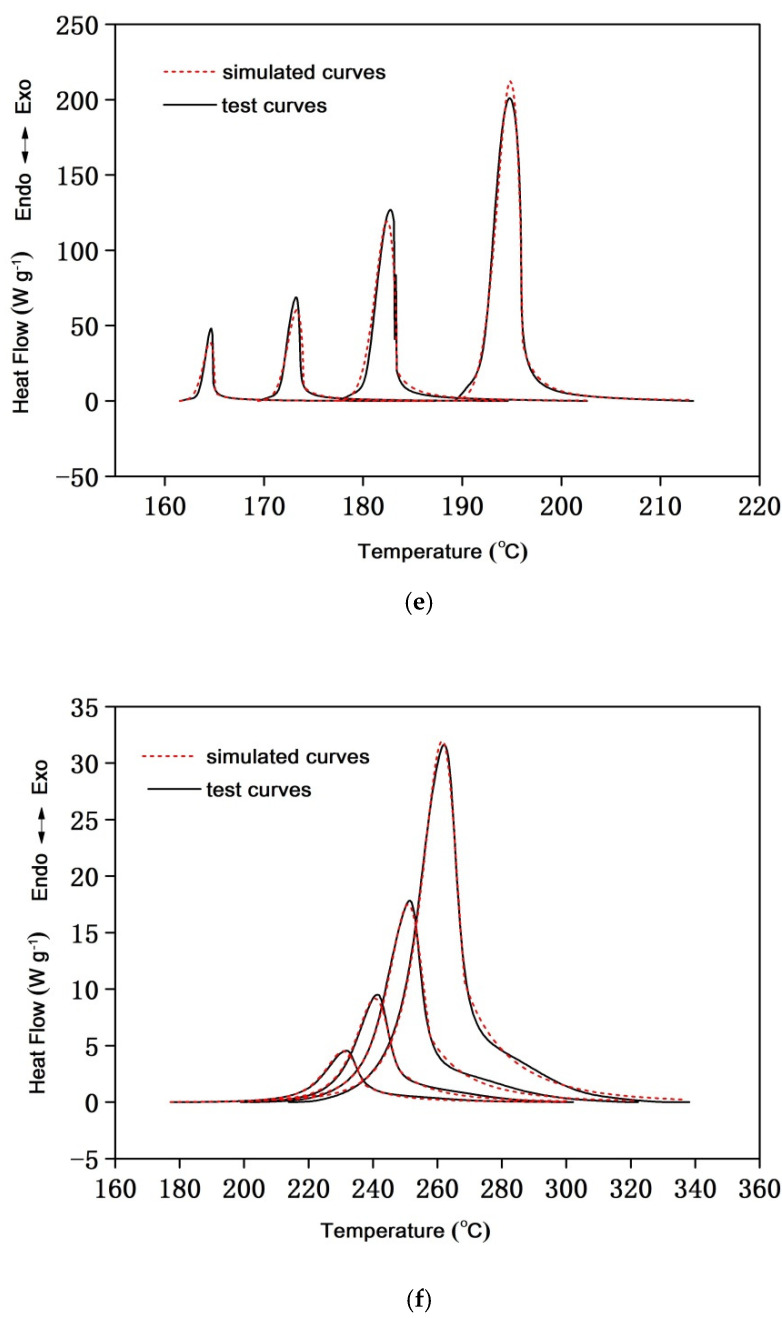
DSC measurements of LLM-116 (**a**) and LLM-226 (**b**) at the heating rates of 2.5, 5, 10 and 20 °C·min^−1^; TG-DTG curves of LLM-116 (**c**) and LLM-226 (**d**); Simulated and test curves of LLM-116 (**e**) and LLM-226 (**f**).

With the raise of the heating rates, the thermal decomposition peaks of both LLM-116 and LLM-226 moved to high temperatures. (Figure 3a,b) To further analyse the thermal decomposition behaviors of LLM-116 and LLM-226, their TG-DTG curves were also studied. (Figure 3c,d) Both of their TG-DTG curves showed a single DTG curve peak. The single peaks indicated that the decompositions are possible to be single-step decomposition reactions; however, they may also be multi-step ones. According to the ICTAC Kinetic committee recommendations, the Multi-Step Model-Fitting method was applied for deep investigations [24]. Based on the analysis of the experimental results of LLM-116 and LLM-226 through the Friedman method, both E and lgA are conversion rate dependent and should be treated as multiple reactions (accelerated reaction type). Based on this, the constructions of the multi-step kinetic models were carried out, and the results showed that the kinetic model of LLM-116 can be regarded as a two-step continuous reaction consisting of BNA (the extended Prout-Tompkins equation) and F2 (second-order) reactions. In contrast, the kinetic model of LLM-226 can be regarded as a two-step continuous reaction consisting of Cn (auto-catalysis n-th order) and F2 reactions. Figure 3e,f are tested, and simulated curves of LLM-116 and LLM-226 are based on experiments and calculations, respectively. For both LLM-116 and LLM-226, the tested curve and the simulated curve are significantly overlapped, indicating that the models match well. The kinetic parameters of each step in the reaction are calculated and shown in Table 1. Based on the comparative research results, the activation energy of the first and initiation reaction of LLM-116 was much less than that of LLM-226, which was accordant with the lower thermal stability of LLM-116 when compared with that of LLM-226 according to the DSC-TG studies.

**Figure 4:**
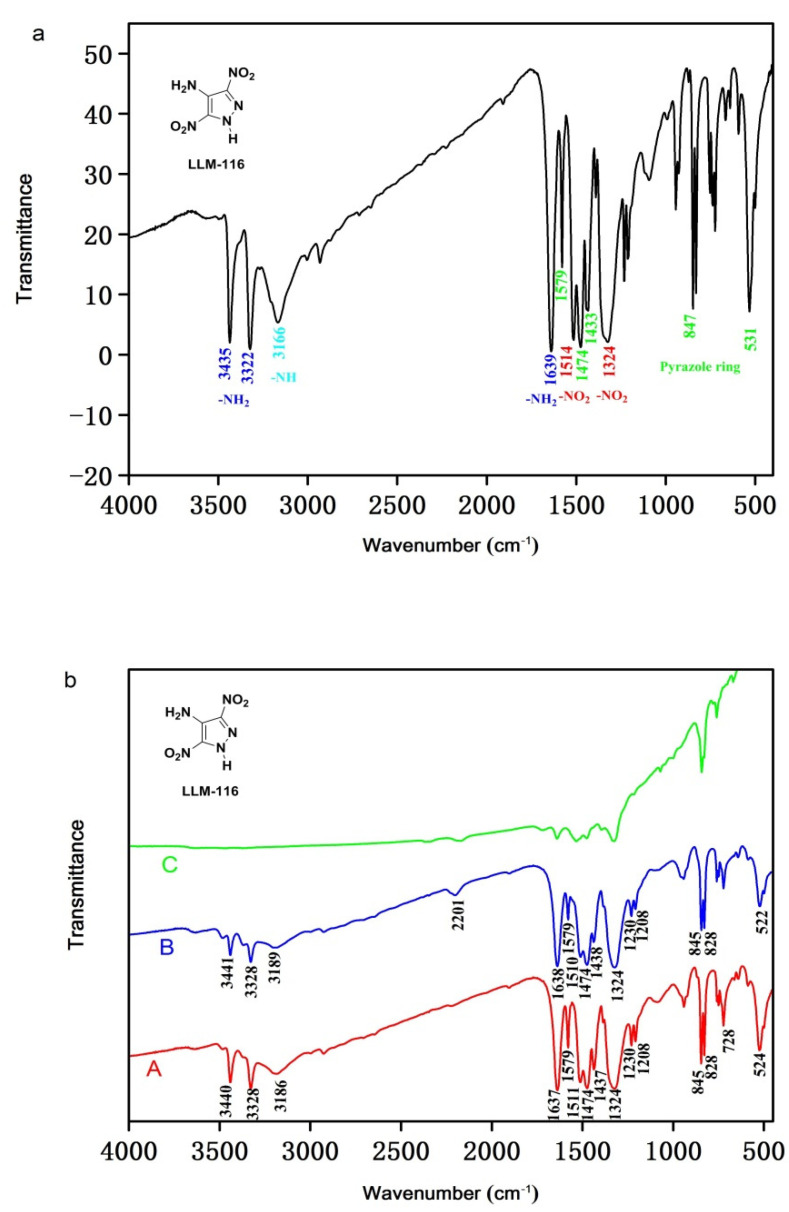

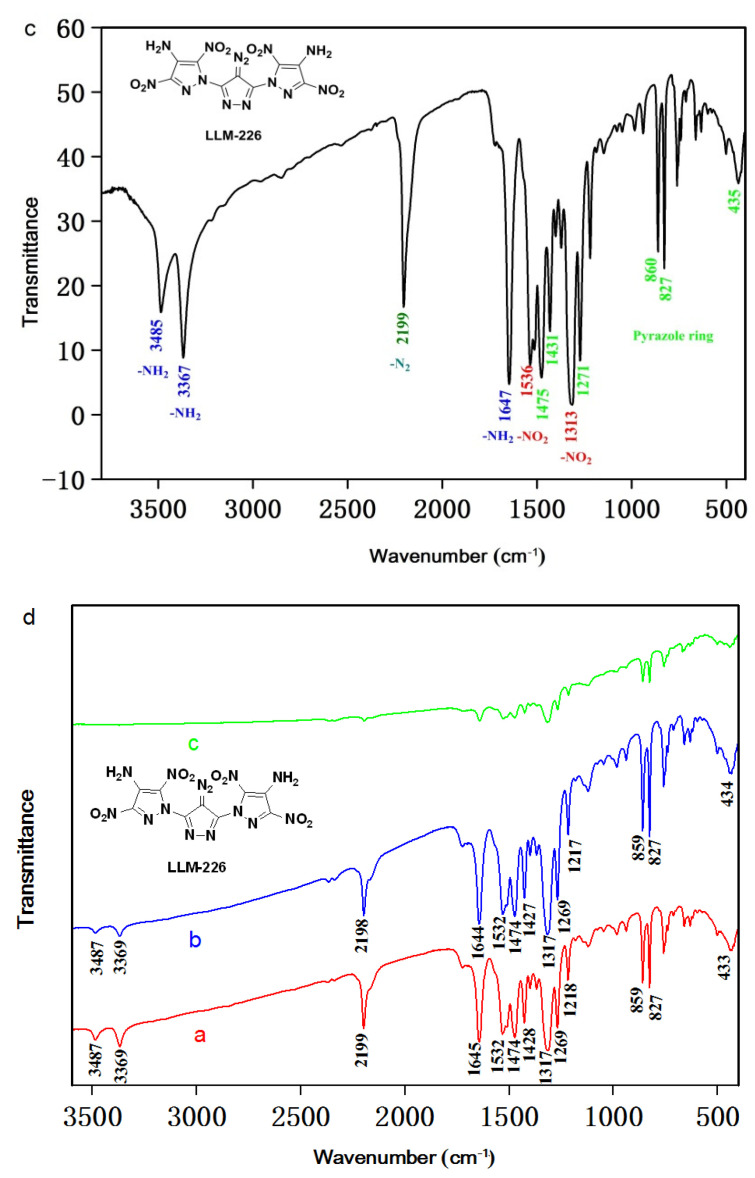
In-situ FTIR spectroscopy of LLM-116 (**a**,**b**) and LLM-226 (**c**,**d**) at different temperatures.

By the experimental results above, although LLM-226 was a trimerization product of LLM-116, their thermal behaviors and properties are very different from each other. For deep analysis of their condensed-phase products during the thermal decompositions, the structural analysis of LLM-116 and LLM-226 were carried out through in situ FTIR spectroscopy under the linear temperature rise condition in real time. (Figure 4) Figure 4a,b showed the FTIR spectrum of LLM-116 at room temperature and heating temperatures of 155 °C, 170 °C, 185 °C. With the raise of the heating temperature, the IR signals of -NH_2_ and -NH moieties of LLM-116 at 3435, 3322 and 3166 cm^−1^ first decreased significantly, while the IR signals for the -NO_2_ (1324 and 1514 cm^−1^) and its molecular skeleton were nearly unchanged. Obviously, the decomposition of LLM-116 started from the -NH_2_ and -NH moieties. Figure 4c,d showed the FTIR spectrum of LLM-226 at room temperature and heating temperatures of 160 °C, 220 °C, 260 °C. Interestingly, the -NH_2_ moieties in the trimerization structure were much more stable than that in LLM-116. Their corresponding IR signals in LLM-226 at 3485 and 3367 cm^−1^ did not decrease quickly ahead of the -NO_2_ (1536 and 1313 cm^−1^) and its molecular skeleton. Although the -N_2_ moiety (with the IR signal located at 2203 cm^−1^) decreased faster than the other parts, it still exhibited a surprisingly superior stability compared to most other diazo-containing energetic structures. Based on the information obtained, it is highly possible that the pyrolysis of LLM-116 started from the –NH moiety, while the pyrolysis of its trimer derivative LLM-226 was likely triggered by the decomposition of the diazo moiety.

To further clarify the corresponding decomposition processes of the similar energetic heterocycles, systematic simulation studies were then carried out. ReaxFF is a method for modeling chemical reactions with atomistic potential based on the reactive force field approach [25], and calculations based on ReaxFF force field were performed with details of the initial reactions discussed to get a deep understanding of the decomposition processes of LLM-116 and LLM-226. The energy of the system was calculated based on Equation (1), in which the $E_{bond}$, $E_{over}$, $E_{under}$, $E_{val}$, $E_{tor}$, $E_{lp}$,$\;E_{Coul}$, and $E_{vdw}$ refer to corresponding bond energy, over-coordination penalty energy, under-coordination correction energy, valence angle energy, torsion angle energy, lone-pair electron energy, Coulomb interaction energies, and van der Waals interaction energy, respectively.

(1) $$E_{system}=E_{bond}+E_{over}+E_{under}+E_{val}+E_{tor}+E_{lp}+E_{Coul}+E_{vdw}$$

As shown in empirical Formula (2), both bond-order formalism and polarizable charge descriptions are applied to investigate the reactive and nonreactive interactions between atoms in this study based on the ReaxFF force field. BO and $r_{ij}$ refer to the distance between atoms *i* and *j*. *P_bo_* terms are empirical parameters. The empirical Formula (2) successfully described all of the σ, π and π−π bonds formed between atoms *i* and *j*. In this study, a standard of BO cutoff of 0.3 [26,27] was chosen, which means that the chemical bond between *i* and *j* atoms was formed if their bond level was greater than 0.3. EEM method [28] is applied to describe the charge of an atom in the calculations based on ReaxFF force field and the description of long-range interactions is added to ReaxFF-lg force field [29] for van der Waals forces. Isothermal-isobaric (NPT) MD simulation with a 0.25 fs time step is also applied to relax the internal stresses and obtain the initial structures of LLM-116 and LLM-226.

(2) $$BO_{ij}=BO_{ij}^{\sigma }+BO_{ij}^{\pi }+BO_{ij}^{\pi \pi }=\exp\left[p_{bo1}\cdot {\left(\frac{r_{ij}}{r_{o}^{\sigma }}\right)}^{P_{bo2}}\right]+\exp\left[p_{bo2}\cdot {\left(\frac{r_{ij}}{r_{o}^{\pi }}\right)}^{P_{bo4}}\right]+\exp\left[p_{bo5}\cdot {\left(\frac{r_{ij}}{r_{o}^{\pi \pi }}\right)}^{P_{bo6}}\right]\;$$

**Figure 5:**
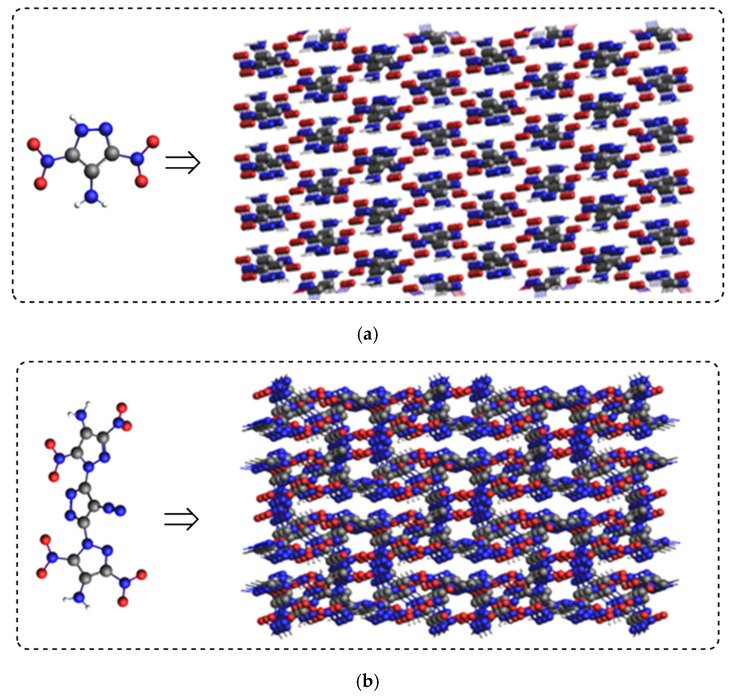
Molecular structures and super cells of LLM-116 (**a**) and LLM-226 (**b**).

The molecular structures and super cells of LLM-116 and LLM-226 were shown in Figure 5. Here, both Berendsen thermostat (100 fs damping constant) and Berendsen barostat (500 fs damping constant) were applied. After a 100 ps of NPT simulation, a 50 ps isothermal isochoric (NVT) MD simulation was carried out with a 0.25 fs time step at 1500 K, 2000 K, and 3000 K, rationally designed high temperatures which could activate the molecules to the desired states instantly for further analysis of the thermal decomposition behaviors of LLM-116 and LLM-226. The thermostat and barostat were same as those applied in the NPT MD simulation, and all MD simulations were carried out using the ADF software package with a reactive force field of ReaxFF-lg. In this study, the bond order cutoff applied to confirm chemical bonds formations was set to 0.3 for all atom pairs and the data calculated from this method was applied to achieve the product distribution and reaction steps. To clarify the initial reaction mechanism which was significant to understand the complete reaction pathway, the first 50 ps decomposition products and pathways of LLM-116 and LLM-226 were studied, respectively. Meanwhile, to understand the effect of temperature on the decomposition products and pathways, their dynamic processes at 1500 K, 2000 K and 3000 K were also determined.

**Figure 6:**
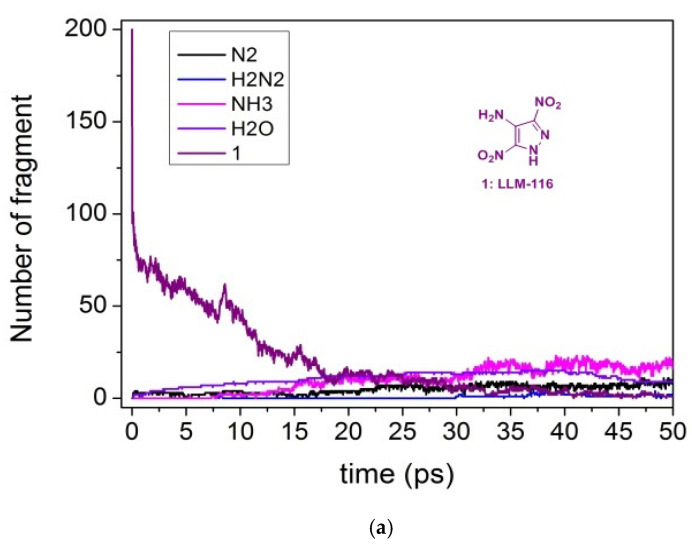

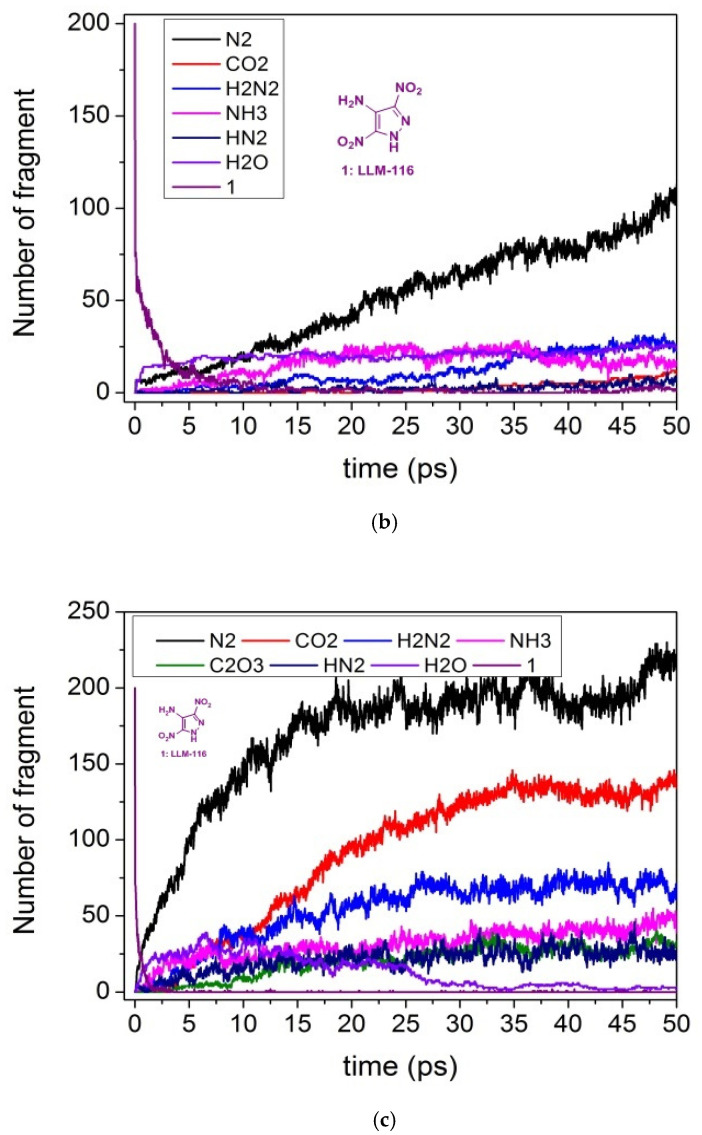
Evolution of key chemical species of LLM-116 (1) at 1500 K (**a**), 2000 K (**b**) and 3000 K (**c**).

**Figure 7:**
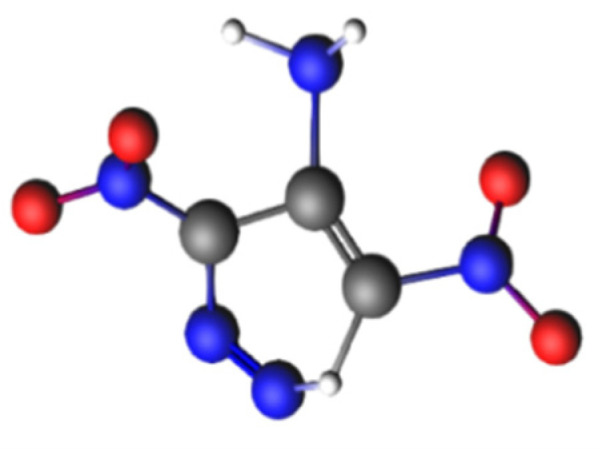
Hydrogen transfer triggers the ring opening of LLM-116.

Figure 6a–c showed the decomposition processes and decomposition products of LLM-116 at 1500 K, 2000 K and 3000 K, respectively. At 1500 K, the main decomposition products were N_2_, H_2_N_2_, NH_3_, H_2_O, and the number of the products was very small compared to 2000 K and 3000 K, which may due to a large number of LLM-116 molecules being not completely decomposed and still retaining their chain-like or cyclic structures. At 2000 K, the amount of nitrogen generated increased significantly. This indicated that a part of LLM-116 molecule was completely decomposed, but the amount of carbon dioxide was still very small, which showed that the carbon chain structure was still not completely decomposed. At 3000 K, the amount of nitrogen generated further increased. Meanwhile, the amount of carbon dioxide generated increased significantly, which indicated that the carbon chain also began to completely decompose. Therefore, as the temperature increases, from the perspective of its different decomposition products, the nitrogen atom in LLM-116 compound completely decomposed first, generating stable products such as nitrogen. As the temperature gradually increased, the carbon chain began to decompose and gave stable products such as carbon dioxide. At 3000 K, the formation of C_2_O_3_ was observed, but this structure was obviously unstable and quickly further decomposed into CO and CO_2_ at high temperatures. Similarly, H_2_N_2_ was also unstable in the external environment and would further decompose. Moreover, it was observed that the initial decomposition of the LLM-116 molecule was mostly caused by intramolecular hydrogen transfer, and Figure 7 showed the intramolecular hydrogen transfer phenomena which resulted in the ring opening of LLM-116.

**Figure 8:**
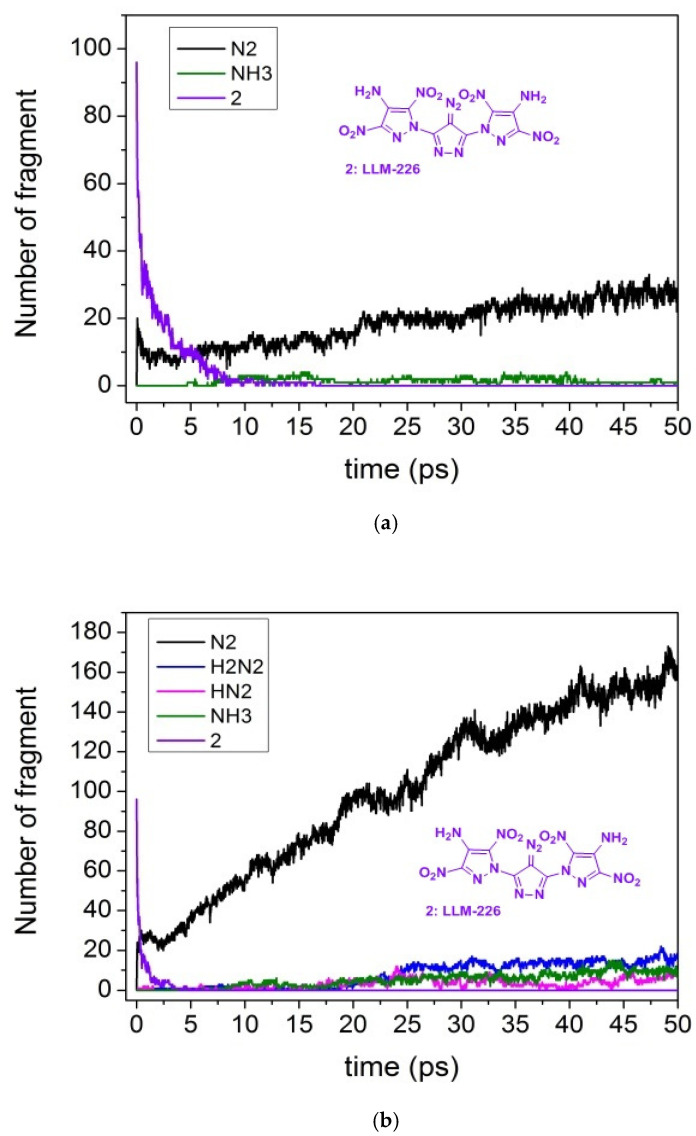

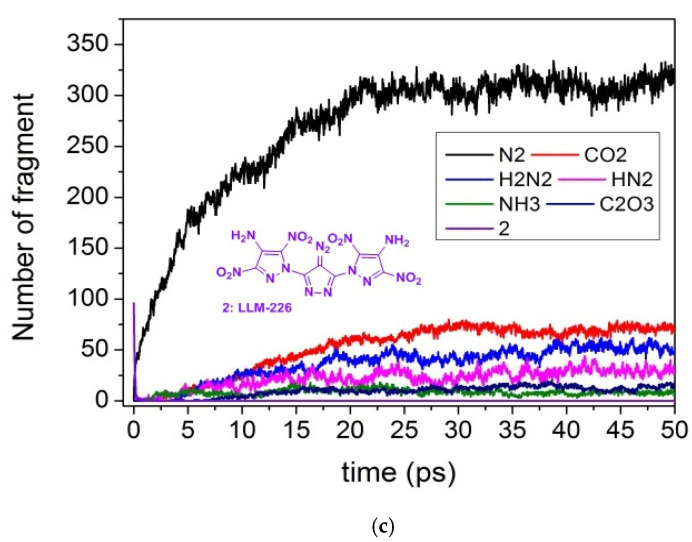
Evolution of key chemical species of LLM-226 (2) at 1500 K (**a**), 2000 K (**b**) and 3000 K (**c**).

**Figure 9:**
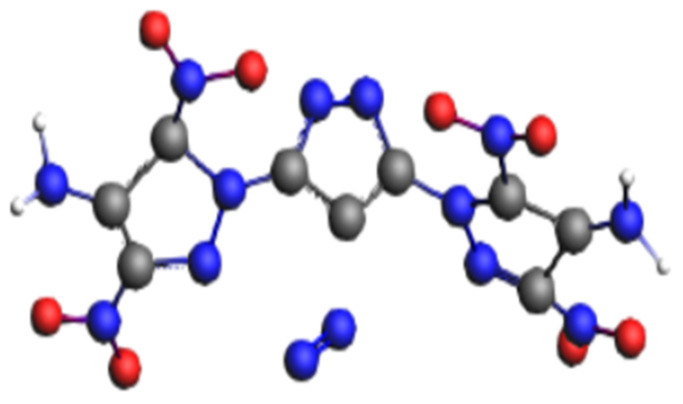
Rupture of C-N bond in the LLM-226 molecule.

Figure 8 shows the decomposition process and decomposition products of LLM-226 at 1500 K, 2000 K and 3000 K. The main decomposition products included N_2_, CO_2_, H_2_N_2_, HN_2_, NH_3_, and C_2_O_3_. Similar to the compound LLM-116, below 1500 K, only a small amount of nitrogen was generated, and the molecular structure was not completely decomposed. When the temperature reached 2000 K, only the amount of nitrogen generated increased significantly, while other small molecule products had only a small increase. When the temperature reached 3000 K, the amount of nitrogen generated continued to increase, but the amount of carbon dioxide generated did not increase significantly, which was different with that of LLM-116. The different results were possibly caused by the lower oxygen content of LLM-226, which made the carbon atoms more difficult to completely decompose through internal redox reactions to form stable products during the pyrolysis process. Moreover, the results showed that the molecular structure of LLM-226 was much more stable than LLM-116, and its initial decomposition was caused by the rupture of carbon-nitrogen bonds at the diazo moiety, which is shown in Figure 9.

**Figure 10:**
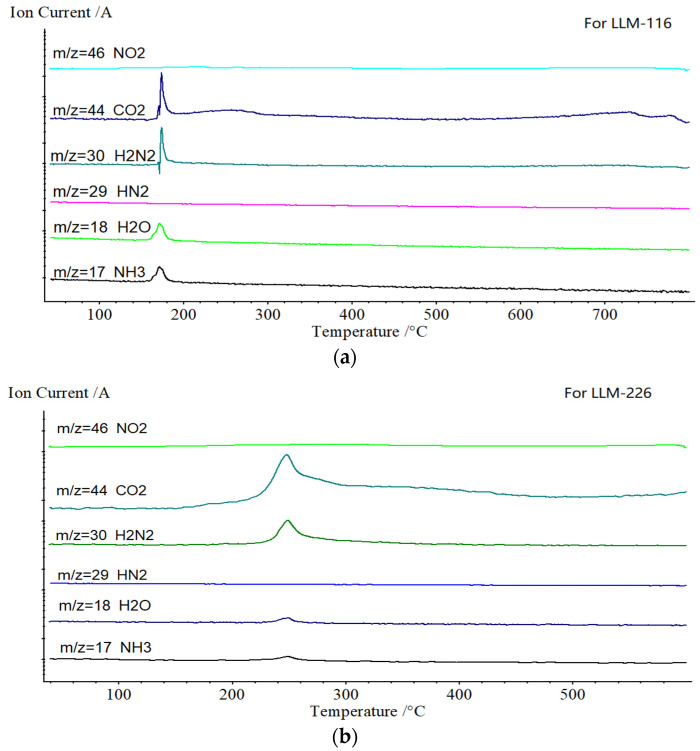
The Mass detections for the decompositions of LLM-116 (**a**) and LLM-226 (**b**).

**Figure 11:**
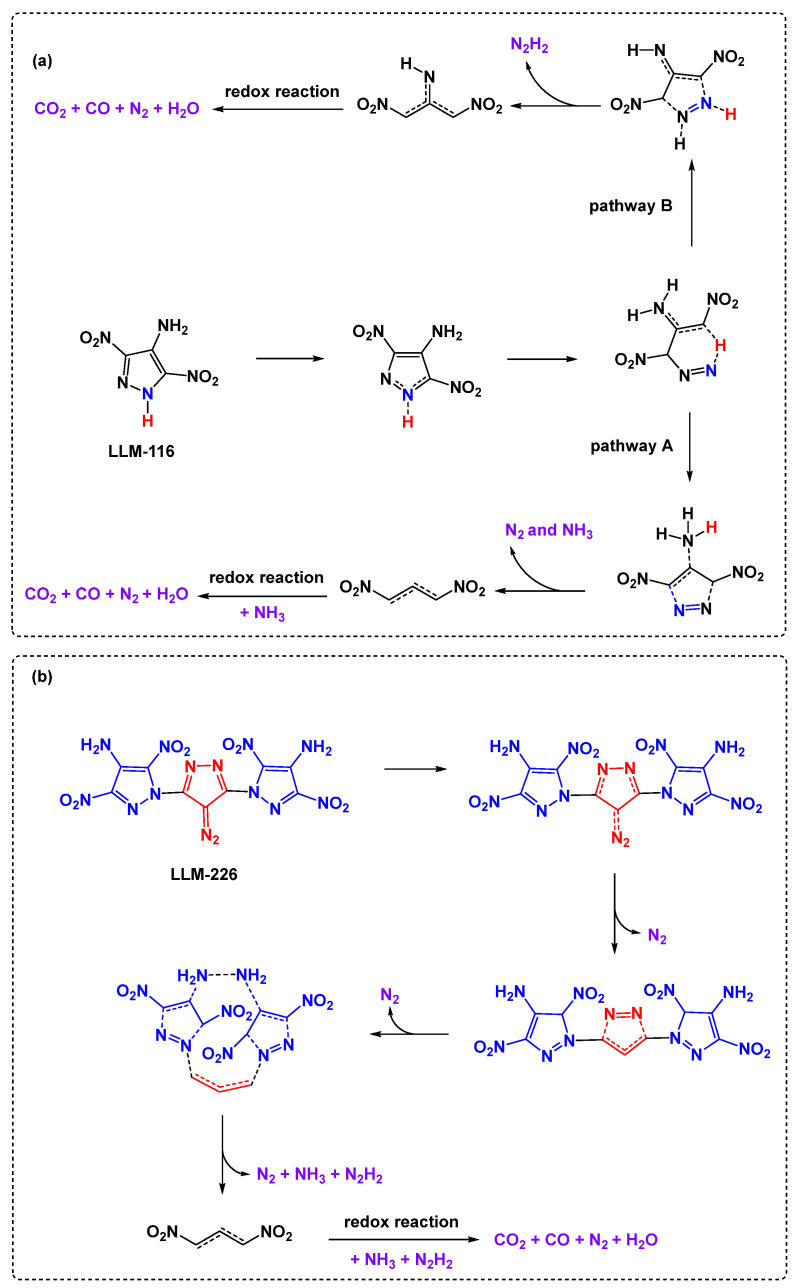
Proposed decomposition mechanisms of LLM-116 (**a**) and LLM-226 (**b**).

There have already been several studies which discussed the possible decomposition mechanisms of energetic pyrazoles under heating conditions. The studies of the Sinditskii group have shown that the splitting-off the nitro group from nitropyrazole structure is a possible way to trigger the decomposition of the energetic molecule, meanwhile, the reaction between conjugated nitro and amine groups may also form fused furazan intermediate, whose ring strains will accelerate further decomposition [30]. The studies from the Kiselev and Muravyev group proposed an impressive thermolysis pathway of 3,5-DNP, commencing with the [1,5] sigmatropic hydrogen shift followed by the consequent molecular elimination of N_2_ and the radical bond scission yielding •NO_2_ [31]. The Prokudin group’s study showed that the thermal decomposition of the nitropyrazole derivatives may start from the intramolecular oxidation of the adjacent carbon atom by the nitro group, which proceeds via a strongly polarized cyclic four-membered transition state. [32] These reported mechanisms from the literature indicate that the pyrolysis of the nitropyrazole derivatives may include different kinds of pathways, such as hydrogen shift, the elimination of N_2,_ as well as internal redox reactions. To further prove the results obtained from the in situ FTIR spectroscopy experiments and calculations based on the ReaxFF force field, the DSC-TG-FTIR-MS quadruple technology [33] was then applied to perform the real-time and continuous analysis of the gaseous products during the pyrolysis of LLM-116 and LLM-226. (Figure 10) According to the experimental mass results obtained, most fragment products detected coincide with the results obtained in the previous sections, except for the fragment of HN_2_, whose signal was not detected. (The signals of fragments of N_2_ and CO were covered by the signal of background nitrogen.) With the combination of all the information obtained, the proposed detailed mechanisms of the thermal decomposition processes of LLM-116 and LLM-226 were demonstrated in Figure 11. For LLM-116, the decomposition started from the hydrogen transfer, which resulted in the formation of the intermediate (in Figure 7) and triggered the ring opening [34]. According to the fragments observed in Figure 10, after the ring opening, NH_3_ could be released from the structure, which also resulted in the elimination of N_2_ through pathway A; in contrast, N_2_H_2_ could be released through pathway B. Activated explosive structures were left after the two pathways, and redox reactions between the fuel moieties (carbon and hydrogen elements) and oxidative moiety (nitro groups) lead to the fragments of CO_2_, CO, N_2_ and H_2_O. (The fragment of C_2_O_3_ was the incompletely oxidized fragment of CO_2_+CO.) Based on the calculation result (Figure 8), the decomposition of LLM-226 was proposed to start from the cleavage of diazo moiety, leading to an activated tricyclic intermediate. The elimination of N_2_ was triggered according to the fragments observed in Figure 10b; moreover, it is possible that the two NH_2_ moieties interact with each other to release the fragments of NH_3_ and N_2_H_2_. A redox reaction between the final remaining fuel elements and oxidative nitro groups in the activated explosive structure could lead to the formation of CO_2_, CO, H_2_O and N_2_. (The fragment of C_2_O_3_ was the incompletely oxidized fragment of CO_2_+CO).

## 3. Methods

**Scheme 1:**
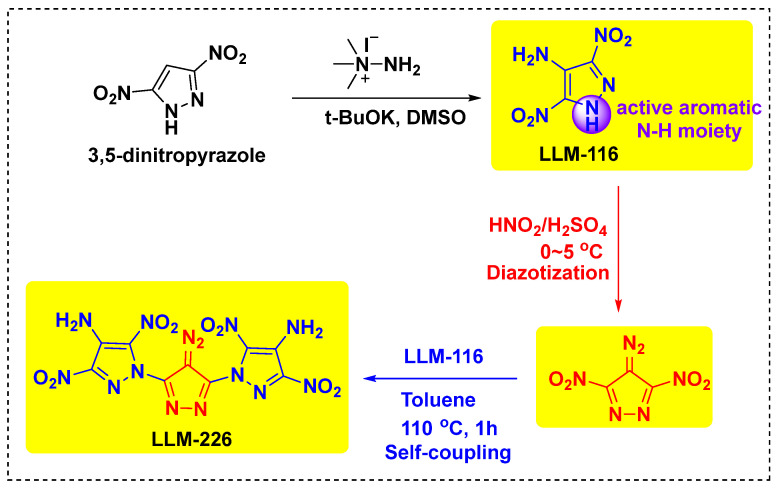
Synthetic route towards LLM-116 and LLM-226.

The samples of LLM-116 and LLM-226 were supplied by Xi’an Modern Chemistry Research Institute. The compounds were synthesized through reported procedures [17,35] (Scheme 1) All the samples were characterized by ^1^H NMR, ^13^C NMR, FTIR and elemental analysis.

The thermal analysis experiments were performed with a model TGDSC STA 449C instrument (NETZSCH, Germany). Operation conditions: sample mass, 0.6 mg; atmosphere, dynamic nitrogen; aluminum cell. The IR spectra were recorded on a Nicolet 60SX FTIR spectrometer employing an HgCdTe detector.

## 4. Conclusions

In summary, comparative thermal behaviors of LLM-116 and its novel trimer derivative LLM-226 were investigated through both experimental and theoretical approaches. Active aromatic N-H moiety exhibited great influence on the corresponding thermal properties and decomposition behaviors. DSC-TG experiment results showed a much higher decomposition peak temperature of LLM-226 with a broader exothermic peak shape, which indicated that the thermal stability was significantly improved after the trimerization, but led to a less concentrated heat release process. Analysis of the condensed-phase systems of the thermal decompositions proved the active aromatic N-H moiety played a key role in the initial process of the decomposition of LLM-116 and the thermal stability of amino groups were improved after the trimerization. Systematic simulation studies based on ReaxFF force field were calculated to further clarify the corresponding decomposition processes of the similar energetic heterocycles, which showed the initial decomposition of the LLM-116 molecule was mostly caused by intramolecular hydrogen transfer, while the initial decomposition of LLM-226 was caused by the rupture of carbon-nitrogen bonds at diazo moiety. The DSC-TG-FTIR-MS quadruple technology was then applied to the analysis of the gaseous products during the pyrolysis of LLM-116 and LLM-226, finding that the experimental result is quite close to the calculation results based on the ReaxFF force field. According to the obtained experimental and calculated results, corresponding mechanisms were finally proposed to clarify the detailed decomposition processes.

## Figures and Tables

**Table 1:** Thermal decomposition kinetic parameters of LLM-116 and LLM-226 obtained through multi-step model-fitting method.

	**Reaction 1 (BNA)**		**Reaction 2 (F2)**	
LLM-116	Activation Energy	82.2	Activation Energy	131.1
	Log(PreExp)	8.7	Log(PreExp)	13.9
	ReactOrder n	0.39	Contribution	0.24
	AutocatOrder	0.79	-	-
	Contribution	0.76	-	-
	**Reaction 1 (Cn)**		**Reaction 2 (F2)**	
LLM-226	Activation Energy	143.6	Activation Energy	120.9
	Log(PreExp)	11.7	Log(PreExp)	10.2
	ReactOrder n	0.65	Contribution	0.42
	Log(AutocatPreExp)	1.2	-	-
	Contribution	0.58	-	-

## Data Availability

All data generated or analyzed during this study are included in this published article.

## Sample Availability

Samples of LLM-116 and LLM-226 are available from the authors.
